# Generation of immune cell containing adipose organoids for in vitro analysis of immune metabolism

**DOI:** 10.1038/s41598-020-78015-9

**Published:** 2020-12-03

**Authors:** Jacqueline Taylor, Julia Sellin, Lars Kuerschner, Lennart Krähl, Yasmin Majlesain, Irmgard Förster, Christoph Thiele, Heike Weighardt, Elvira Weber

**Affiliations:** 1 Biochemistry and Cell Biology of Lipids, Life and Medical Sciences Institute (LIMES), University of Bonn, Carl-Troll-Straße 31, 53115 Bonn, Germany; 2 ZSEB, Center for Rare Diseases, University Hospital Bonn, 53127 Bonn, Germany; 3 Immunology and Environment, Life and Medical Sciences (LIMES) Institute, University of Bonn, Carl-Troll-Straße 31, 53115 Bonn, Germany; 4 Present Address: Division Vascular Signaling and Cancer (A270), German Cancer Research Center (DKFZ), 69120 Heidelberg, Germany; 5 Present Address: Faculty of Biosciences, University of Heidelberg, 69120 Heidelberg, Germany

**Keywords:** Biological techniques, Cell biology, Immunology

## Abstract

Adipose tissue is an organized endocrine organ with important metabolic and immunological functions and immune cell-adipocyte crosstalk is known to drive various disease pathologies. Suitable 3D adipose tissue organoid models often lack resident immune cell populations and therefore require the addition of immune cells isolated from other organs. We have created the first 3D adipose tissue organoid model which could contain and maintain resident immune cell populations of the stromal vascular fraction (SVF) and proved to be effective in studying adipose tissue biology in a convenient manner. Macrophage and mast cell populations were successfully confirmed within our organoid model and were maintained in culture without the addition of growth factors. We demonstrated the suitability of our model for monitoring the lipidome during adipocyte differentiation in vitro and confirmed that this model reflects the physiological lipidome better than standard 2D cultures. In addition, we applied mass spectrometry-based lipidomics to track lipidomic changes in the lipidome upon dietary and immunomodulatory interventions. We conclude that this model represents a valuable tool for immune-metabolic research.

## Introduction

Physiologically relevant cell based assays are of great importance for translational biological research. To overcome the limitations of classical 2D culture, including disturbances in cell shape or polarity, insufficient cell–cell contacts or reduced extracellular matrix (ECM) synthesis^1,2^, recent research has become more focused on developing more physiological cell culture systems. These revelations have sparked greater need for the development of 3D cell culture systems^3^. 3D models are of importance for tissue engineering, drug discovery, but also provide models for cancer research, organ development or understanding disease pathologies^4–6^.The advantages of 3D culture include faster and cost-efficient drug safety and efficacy studies^7^, as well as improved prediction of drug resistance which is more comparable to the in vivo situation^8^. 3D models can be divided into two classifications—scaffold-based or scaffold-free systems. A scaffold can be composed of synthetic or biological material, forming hydrogels or membranes^4^. Scaffold-based systems provide a better base for the architecture of 3D structures and are needed for some cell types that are unable to aggregate without. However, the 3D structures which form are often irregular in size, the scaffold can absorb test compounds and spheroids can absorb factors included in the scaffold which can influence the metabolism of the spheroids. In a scaffold-free culture, cells grow in a more natural environment, allowing for self-assembly of cells into a more native 3D structure without the influence of external physical cues that possibly block drug or growth factor delivery. Various scaffold-free techniques have been described, including hanging droplets, magnetic levitation or ultra-low attachment (ULA) plates^9–15^. The use of ULA plates for 3D cultures present several advantages, including their ease of handling, low costs and high reproducibility of spheroid growth.

The development of 3D models which mimic adipose tissue is already underway^16^. Initially, 3D adipose tissue models were composed of the murine adipocyte precursor cell line, 3T3-L1, grown using a scaffold-based system^17,18^. Spheroids were cultured with or without the addition of other cell types such as macrophage or endothelial cell lines^17–20^. More recently, spheroids have been grown from primary cells derived from the stromal vascular fraction (SVF)^21^. The SVF is highly heterogeneous and contains an array of cell types including adipose tissue derived stem cells (ASC) as well as other adipocyte precursors^22–25^ which have the ability to differentiate into adipocytes. Scaffold-free 3D adipose tissue spheroid models have been generated using 3T3-L1 cells^26^ or primary SVF using magnetic levitation^19^, hanging drop cultures^12^ or ULA plates^13,27^. Muller et al. used human SVF to generate spheroids which mirrored the cellular architecture of adipose tissue. They observed that aside from adipocytes, an endothelial cell population was maintained in SVF adipose spheroids and defined them as organoids^13^. Organoids are ‘mini’ organs, consisting of tissue relevant cell types derived from progenitor cells grown in 3D in vitro^28,29^. In accordance with the definition from Lancaster and Knoblich, organoids must fulfill the following criteria: (1) it must contain multiple organ-specific cell types; (2) it must be capable of recapitulating some specific organ function; and (3) its cellular components must be spatially organized similar to an organ^28^. Mini organs such as guts, livers, hearts and even brains have already been successfully developed^28–32^.

Metabolic syndrome is a common metabolic disorder, which clusters conditions such as obesity, insulin resistance, hypertension and hyperlipidemia, and increases the risk of cardiovascular diseases, stroke and type 2 diabetes. These metabolic disorders are associated with adipose tissue dysfunction^33^. In addition, obesity has been correlated with the risk of various cancer types^5^. Adipose tissue responds to insulin by inhibiting lipolysis and increasing lipid storage leading to adipocyte expansion. During both obesity and type 2 diabetes, insulin resistance can occur^6^. Importantly, adipose tissue also regulates endocrine and immunological functions by secreting hormones, adipokines and cytokines^34–36^. Local immune cells, in particular macrophages, play an important role in fat tissue biology^37,38^. Resident adipose tissue macrophages (ATM) are important for the development of adipose tissue^39^, regulation of adipocyte hypertrophy, lipid storage and removal of dead adipocytes^36^. ATM take up and clear triglycerides and non‐esterified fatty acids released by hypertrophic insulin‐resistant adipocytes^40^. Such examples of adipocyte-macrophage crosstalk emphasize the need for different cell types to be considered when studying adipose tissue biology. Furthermore, there is growing importance in studying lipid metabolism and turnover during homeostasis and disease using physiologically relevant models.

Here we describe the generation of self-organizing scaffold-free 3D adipose tissue organoids from primary SVF cells using methodology which can be easily implemented in any laboratory. ULA plates were used to produce SVF-containing spheroids. Adipocyte precursor cells within spheroids were differentiated into mature adipocytes in the presence of immune cells. Immune cells were maintained without the external supplementation of growth factors. Our adipose tissue organoid model is the first which has been used to monitor changes in the lipidome using MS-based technology. We could show that cultures were capable of recapitulating characteristic features of white adipose tissue better than 2D cultures. Using this approach, we have demonstrated that this organoid system is a more physiologically relevant model for in vitro studies aimed at observing immune-adipocyte crosstalk and the influence of metabolic, dietary or inflammatory interventions on adipose tissue lipid metabolism.

## Results

### Primary stromal vascular fraction self-organizes into a 3D adipose tissue spheroid

**Figure 1:**
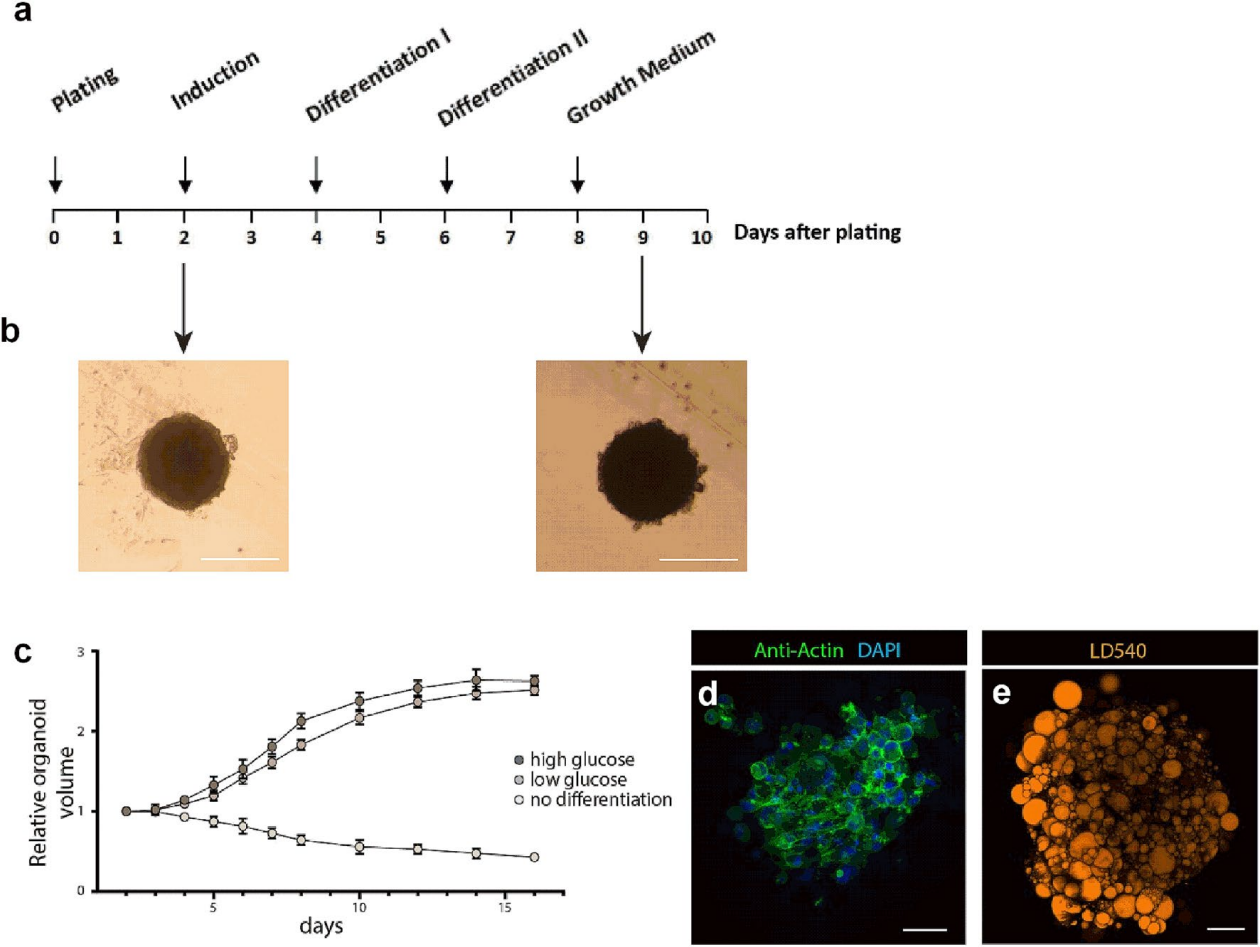
Primary stromal vascular fraction self-organizes into a 3D adipose tissue spheroid. (**a**) Time line of differentiation of adipose tissue spheroids after plating in 3D. (**b**) Representative figures of spheroids after plating in 3D (bar 150 µm). (**c**) Volume of spheroids was analyzed with and without differentiation cocktail in low and high glucose DMEM over 16 days [n = 48 spheroids (technical replicates) for each condition from 2 individual experiments (biological replicates)]. Differentiated spheroids were stained with (**d**) DAPI (blue) and ActinGreen or (**e**) LD540 (bar 50 µm).

Unlike subcutaneous adipose tissue (SAT), visceral adipose tissue (VAT) is more prone to inflammation and immune cell infiltration, and is a greater contributor to the development of insulin resistance^41^. Because of its inflammatory potential, VAT plays a major role in the development of metabolic diseases such as obesity and diabetes. To generate 3D visceral adipose tissue spheroids, we decided to use scaffold-free conditions to exclude the potential influence of artificial scaffolds on cells and to allow cells to synthesize their own ECM as previously shown by Kapur et al. and Amos et al.^42,43^. For this, visceral adipose tissue was isolated from healthy, lean male mice and digested to obtain the SVF. SVF were cultured in 2D until confluency before seeding 50,000 cells per well into ULA plates^42,44^. Cells were cultured to allow for self-organization into spheroids^18,42,44^. After 48 h, adipocyte differentiation was induced using a standard adipocyte differentiation cocktail consisting of insulin, dexamethasone, rosiglitazone and 3-isobutyl-1-methylxanthin (IBMX) (Fig. 1a). 48 h later, spheroids were cultured in growth medium containing only insulin. From day 9 onward, spheroids were considered fully mature (Fig. 1a). To efficiently monitor the differentiation of 3D adipose spheroids, the volume of the spheroids was assessed daily during the differentiation process up to day 16 after spheroid formation (Fig. 1b,c). The relative increase in spheroid volume during adipocyte differentiation was assessed by quantitative microscopy using a CellProfiler-based method^45^. Spheroids cultured without the addition of adipogenic reagents served as a negative control (Fig. 1c). The volume of spheroids increased from day 4 and reached a plateau on day 12 irrespective of the glucose concentration in the cultivation medium. This increase in volume is not due to proliferation of cells in the organoid (Supplementary Fig. S1) but may be partially attributed to an expansion in lipid droplet storage, which profoundly increased in size as revealed by microscopy (Supplementary Fig. S1). Differentiated spheroids stained with LD540 contained adipocytes with large unilocular lipid droplets, confirming efficient adipocyte differentiation (Fig. 1e). In addition, adipocyte differentiation was confirmed by a clear upregulation of the adipocyte marker genes pparγ, adiponectin and fabp4 by qPCR analysis (Supplementary Fig. S1). Furthermore, staining with Actin Green showed that spheroid cells formed an organized network (Fig. 1d).

### Mass spectrometry-based lipid analysis of 3D adipose tissue spheroids is a new tool to efficiently study lipid metabolism in vitro

**Figure 2:**
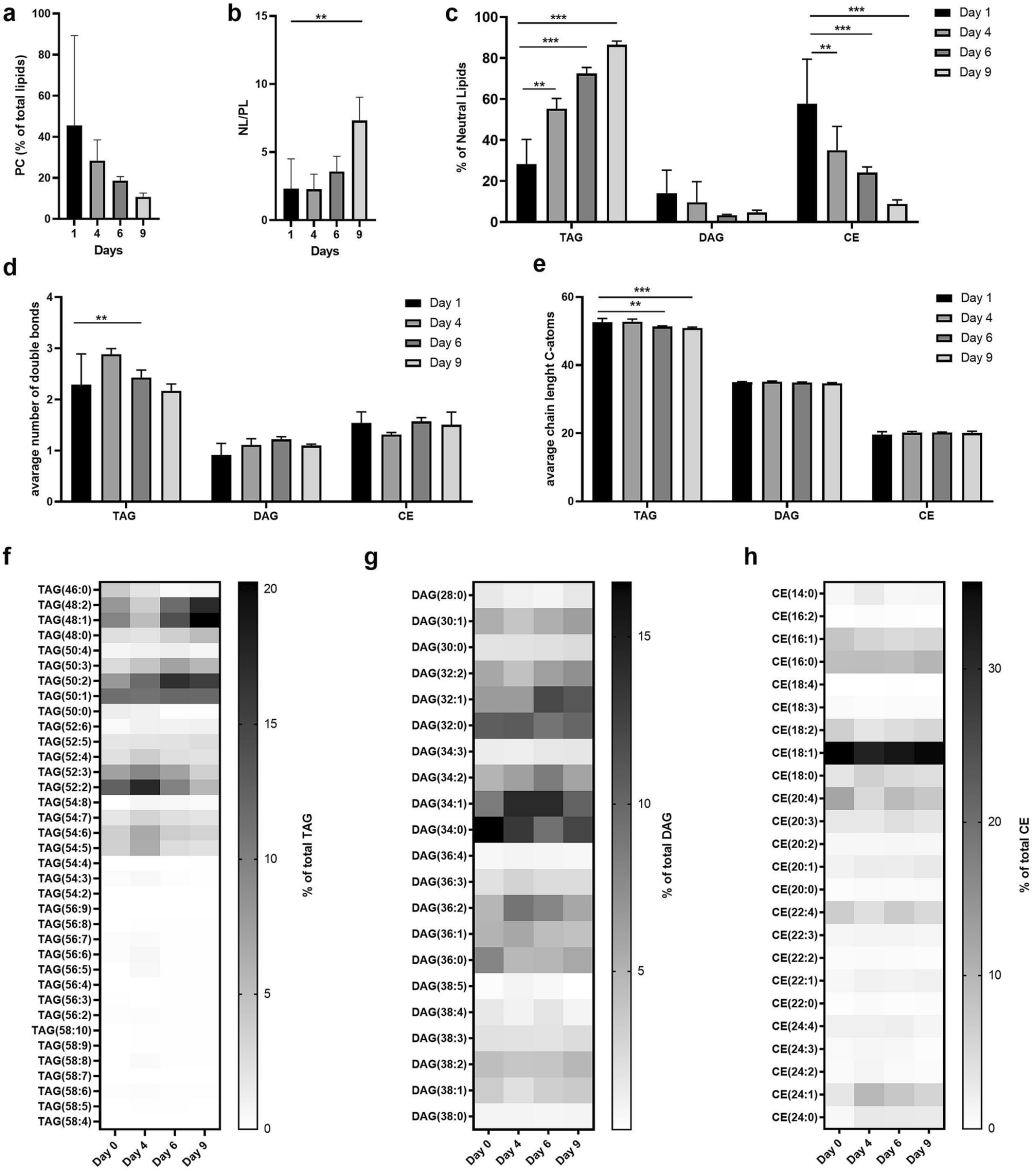
Mass spectrometry lipid analysis of 3D adipose tissue spheroids is a new tool to efficiently study lipid metabolism in vitro. SVF were differentiated and harvested at day 1, day 4, day 6 and day 9. Total lipids were extracted and analysed by MS. (**a**) Amount of PC normalized to the amount of total lipids. (**b**) Ratio of NL to polar lipids. (**c**) Composition of NL. (**d**) Average number of double bonds. (**e**) Average chain lengths of NL. (**f**) Heatmap of TAG species as % of total TAG amount. (**g**) Heatmap of DAG species as % of total DAG amount. (**h**) Heatmap of CE species as % of total CE amount (n = 3 individual experiments (biological replicates) each performed in technical triplicates). Data are presented as mean ± SD. Significance was determined using Two Way ANOVA with Dunette correction for multiple comparisons. *p < 0.05, **p < 0.01 and ***p < 0.001.

Maturing adipocytes store lipids mainly as triglycerides (TAG) in lipid droplets. To monitor lipidomic changes during differentiation, spheroids were analyzed for changes in lipid composition by mass spectrometry (MS). Lipidomic analysis not only identifies and quantifies different lipid classes but also enables the discovery of their functions and their association within metabolic pathways^46,47^. SVF was cultured and differentiated as described above. Spheroids were harvested at day 1 (24 h after seeding), day 4 (2 days after the addition of the adipogenic cocktail), day 6 (spheroids growing in insulin containing media i.e. differentiation media) and day 9 (24 h after changing from differentiation media to non-insulin containing medium). Total lipids were extracted and analyzed by MS. The two most abundant lipid classes observed were phosphatidylcholine (PC), the main component of membranes, and TAG. TAG is the major storage lipid of adipocytes, and forms the lipid droplet core along with other neutral lipids (NL)^48^. During adipocyte differentiation, the ratio of PC to total lipids declined over time (Fig. 2a). Next, the analyzed ratio of NL (CE, DAG, TAG) to all other analyzed lipid species (Cer, LPC, LPE, PC, PE and PG), hereafter referred to as polar lipids (PL), was compared. The ratio of NL to PL increased during differentiation (Fig. 2b), possibly reflecting the decline in the ratio of PC. A detailed analysis of various NLs revealed that the proportion of TAG, which is the most important storage form of fatty acids, increased over time while the fraction of cholesterol esters (CE) decreased during differentiation (Fig. 2c). Furthermore, changes in saturation (Fig. 2d) and carbon chain length (Fig. 2e) of different NL classes could be detected. A decrease in TAG chain length and an increase in saturation levels were observed over time. DAG and CE also showed a significant difference in carbon chain length but not in the frequencies of double bonds (Fig. 2d,e). In heat maps depicted in Fig. 2f–h the distribution of specific TAG, DAG or CE species is shown. The most prominent observations were an increase in TAG species (48:2) and (48:1) in fully differentiated organoids (Fig. 2f), a wide distribution of several species of DAG (Fig. 2g), and a dominance of CE (18:1) throughout the whole differentiation process (Fig. 2h). Taken together, these lipidomics data demonstrate that this spheroid model in combination with MS analysis is useful to study lipid changes during adipose differentiation in vitro.

### Lipid composition of SVF derived spheroids resembles in vivo adipose tissue

**Figure 3:**
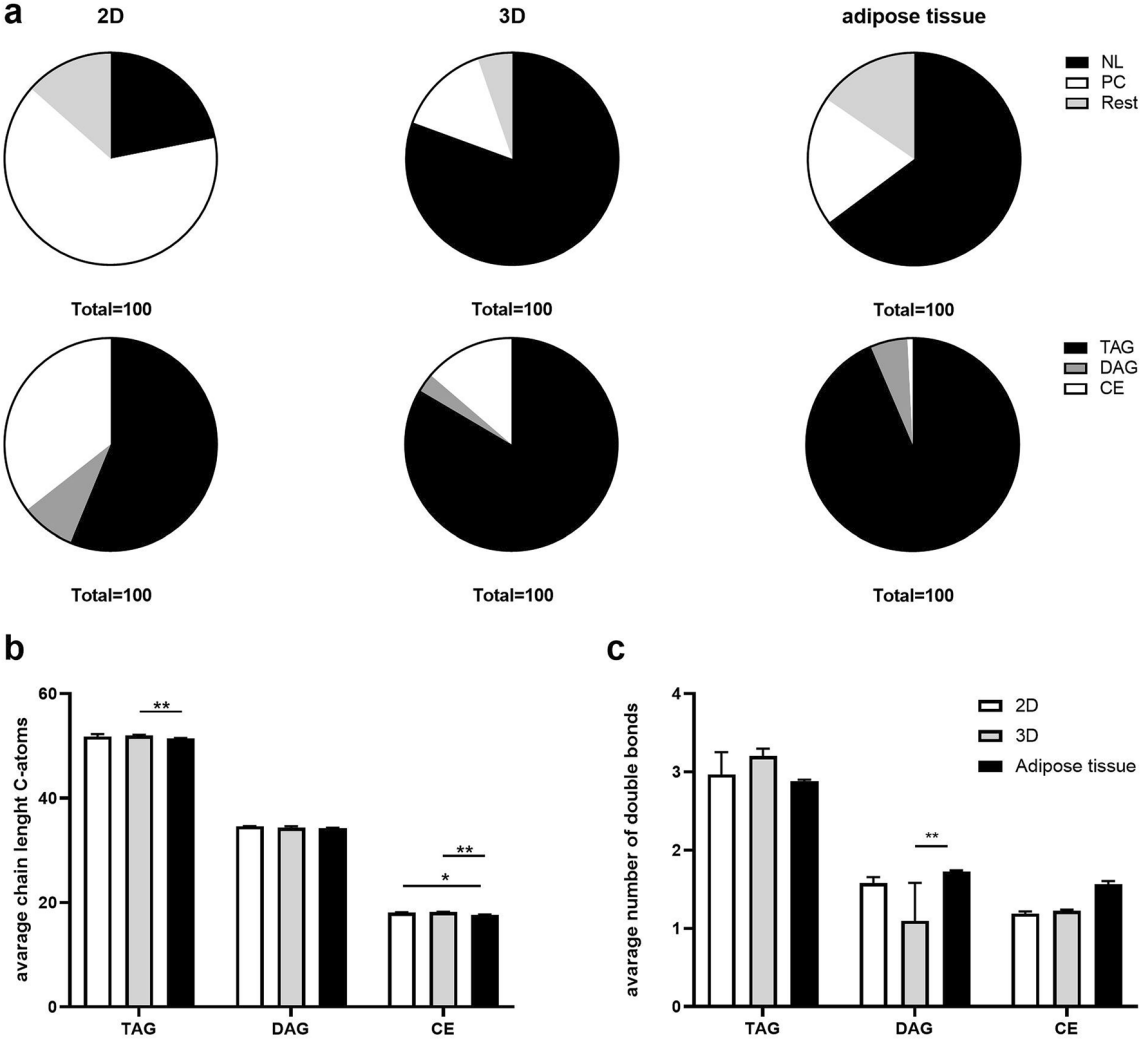
Lipid composition of SVF derived spheroids resembles in vivo adipose tissue. Mouse SVF were cultured under 2D or 3D conditions, when fully differentiated lipids were isolated and analysed by MS. Profiles were compared with lipid profiles of freshly isolated mouse adipose tissue. (**a**) Ratio of neutral lipids to PC and rest and composition of NLs. (**b**) Average chain length of C-atoms of NLs. (**c**) Average number of double bonds of NLs. (n = 3 individual experiments (biological replicates) each performed in technical triplicates). Data are presented as mean ± SD. Significance was determined using two way ANOVA with Dunette correction for multiple comparisons. *p < 0.05, **p < 0.01 and ***p < 0.001.

Turner et al. and Klingelhutz et al. could show that adipose spheroids have a higher expression of adipocyte differentiation markers such as PPARγ compared to 2D cultures^12,18^. Next we compared lipid profiles of differentiated SVF cells grown in 2D and 3D for 9 days to lipid profiles of freshly isolated visceral mouse adipose tissue (Fig. 3a). In all samples, PC and NL were the most abundant lipid classes. Turner et al. showed an increased TAG content in a 3D culture of 3T3-L1 spheroids compared to 2D cultures^18^. Consistent with this observation, frequencies of NL in 3D differentiated SVF were enhanced compared to those in 2D cultures and were broadly similar to levels in adipose tissue. In addition, the frequency of PC in spheroids was largely comparable to adipose tissue, while 2D cultures exhibited an increased PC fraction (Fig. 3a upper panel), pointing to profound differences in the distribution of lipid classes when comparing 2D cultures and 3D spheroids with ex vivo adipose tissue. In line, adipocytes cultured in 2D develop several small lipid droplets per cell (multilocular), while adipocytes grown in 3D cultures are able to form one large lipid droplet similar to monolocular adipocytes in adipose tissue^12,49^.This change in lipid droplet size leads to an altered surface to volume ratio of the organelle. Indeed, adipose tissue NLs were comprised of approximately 90% TAG compared to 50% in 2D cultures (Fig. 3a lower panel). The NLs of the spheroids consisted of nearly 75% TAG and thereby closely mimic the in vivo situation. However, only slight differences in average carbon chain length or double bond count in NLs were detected between the three sample types (Fig. 3b,c). These data demonstrate that the lipidome of 3D spheroids largely resembles that of adipose tissue and point to the ability of spheroids to regain the shape and the lipid composition of in vivo adipose tissue.

### 3D adipose tissue spheroids can be used to monitor either dietary- or insulin-induced metabolic changes in the lipidome

**Figure 4:**
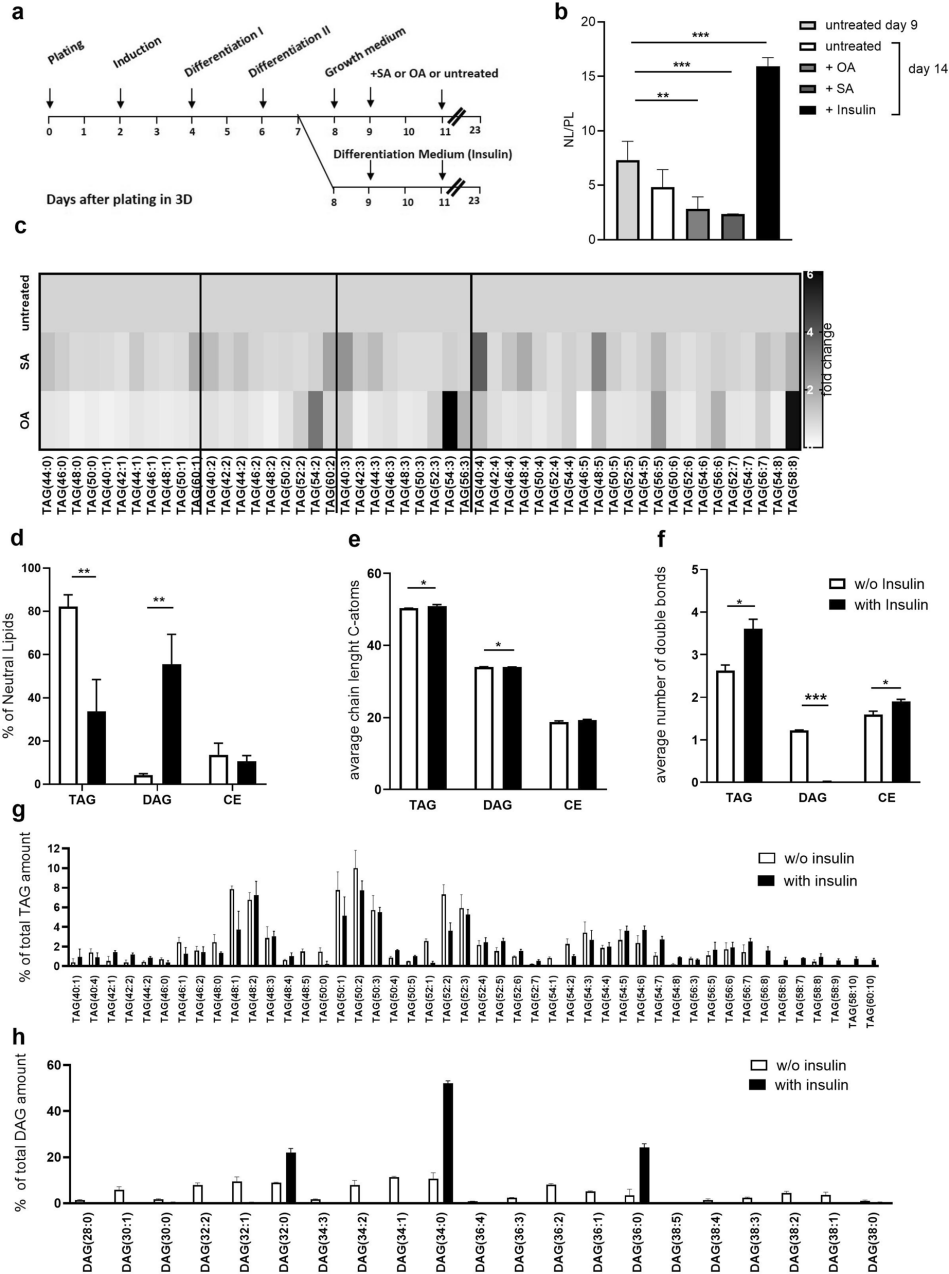
3D-adipose tissue spheroids can be used to monitor either dietary- or insulin-induced metabolic changes in the lipidome. Differentiated SVF were treated for 14 days with OA, SA or Insulin. Total lipids were extracted and analysed by MS. (**a**) Experimental timeline. (**b**) Ratio of NL to polar lipids. (**c**) Fold change of TAG species. (**d**) Composition of neutral lipids. (**e**) Average chain lengths of neutral lipids. (**f**) Average number of double bonds. (**g**) TAG and (**h**) DAG species as percentage of total TAG and DAG (n = 3 individual experiments (biological replicates) each performed in technical triplicates). Data are presented as mean ± SD. Significance was determined using two way ANOVA with Dunette correction for multiple comparisons. *p < 0.05, **p < 0.01 and ***p < 0.001.

High loads of saturated or unsaturated fatty acids have been previously proven to exert different biological impacts on adipose tissue^50,51^. For example, unsaturated fatty acids showed higher oxidation rates and are released as free fatty acids^52^, while saturated fatty acids accumulate as DAG, which desensitizes cells to insulin stimulation^53,54^. Next, we studied the influence of these molecules on adipocyte lipid content. To exemplify this, oleic acid (OA), a mono-unsaturated omega-9-fatty acid, and saturated stearic acid (SA) were chosen. The effect of additional insulin supplementation on spheroid lipid metabolism was also studied. Mouse SVF cells were cultured as spheroids and differentiated for 9 days. Subsequently, they were treated every 48 h with insulin (5 µg/mL), 0.5 mM OA, 0.5 mM SA or left in growth medium without additives for an additional 14 days. Lipid content was then examined by MS (Fig. 4a). In spheroids incubated in growth medium without any additives, the ratio of NL-to-PL decreased over time. Interestingly, OA and SA treatment further decreased the ratio, likely by inducing lipolysis caused by the long-term treatment of and excessive amounts of fatty acids (Fig. 4b). However, insulin supplementation increased the content of NL relative to PL. To analyze this in more detail, the composition of different TAG species was compared upon treatment with either SA, OA or the untreated control (Fig. 4c). No obvious differences were observed in SA-treated spheroids, while in OA-treated spheroids, two distinct polyunsaturated TAGs [TAG (54:3) and (58:8)] increased in abundance. Upon insulin treatment, an increase in the DAG-fraction and a concomitant decrease in the TAG-fraction within the NL population was observed (Fig. 4d). The carbon chain length of TAG and DAG increased after insulin supplementation (Fig. 4e), and significantly more unsaturated TAG species could be observed, while DAG species appeared to be more saturated (Fig. 4f).To further identify which species are affected, the frequencies of each individual TAG and DAG species were determined (Fig. 4g, h). While insulin treatment was only able to induce a small magnitude of change in the quantities of individual TAG species, an increase in the whole TAG pool could be observed. In contrast, the broad range of different DAG species became very narrow in spheroids upon treatment with insulin. Spheroids were composed of only three DAG species, (32:0), (34:0) and (36:0), upon insulin treatment (Fig. 4h).

### Adipose tissue spheroids contain immune cells

**Figure 5:**
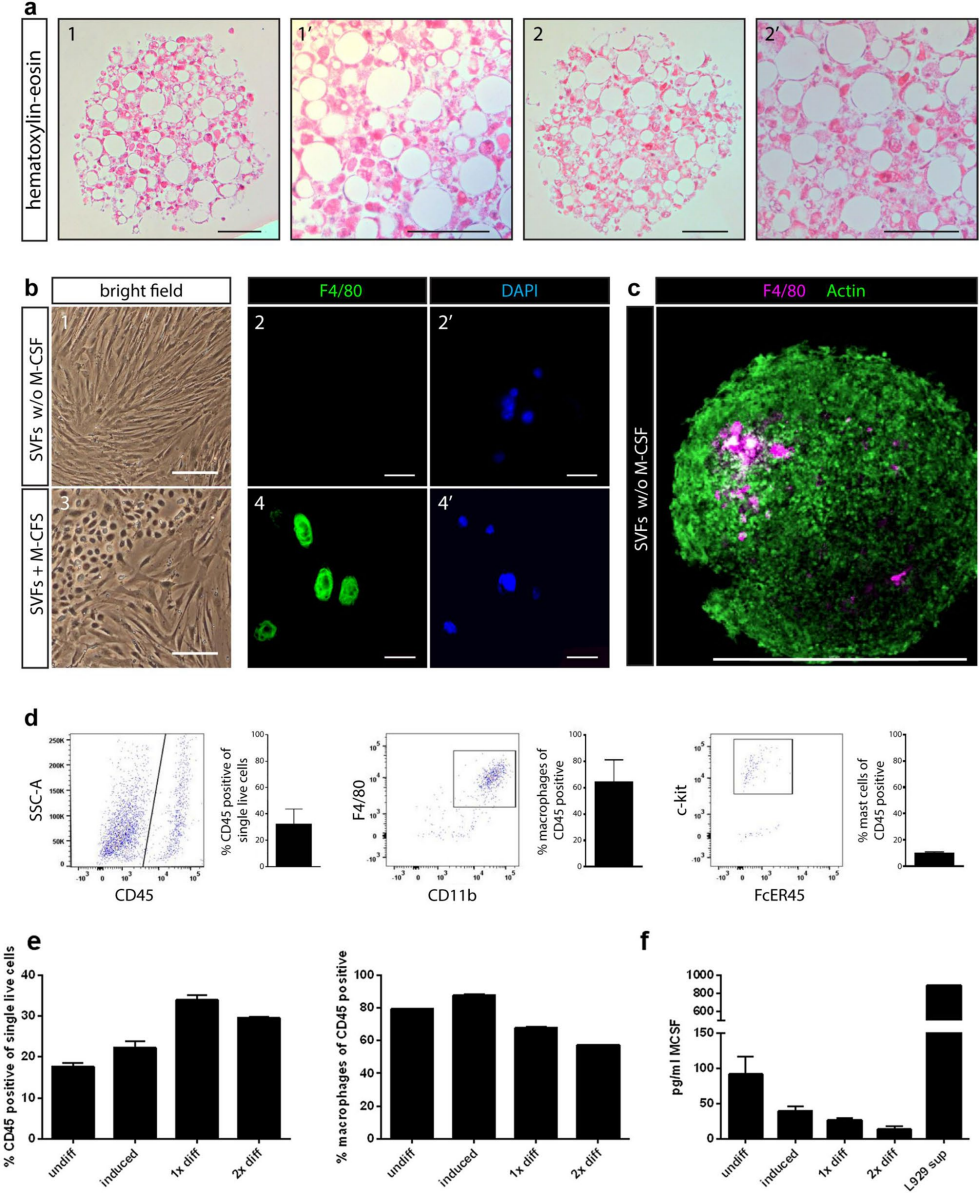
Adipose tissue spheroids contain immune cells. (**a**) H&E stained sections of different layers of JB4 embedded spheroids (bar 50 µm). (**b**) 1&3: brightfield microscopy of confluent 2D cultures of SVF cells with and without M-CSF (bar 100 µm). 2&4: F4/80 (green) and DAPI (blue) staining of subconfluent SVF 2D cultures with and without M-CSF (bar 20 µm). (**c**) Differentiated SVF spheroid stained with F4/80 (purple) and ActinGreen (bar 150 µm). (**d**) Differentiated SVF spheroids were analysed by flow cytometry (n = 3–6 independent experiments (biological replicates) each performed in technical triplicates). (**e**) Frequencies of CD45 positive cells (left) and macrophages (right) in undifferentiated, induced and differentiated spheroids (n = 3 individual experiments (biological replicates) each performed in technical triplicates). (**f**) M-CSF was measured in supernatants of undifferentiated, induced and differentiated spheroids (n = 2–4 individual experiments (biological replicates) each performed in at least technical triplicates).

As the SVF is made up of a heterogeneous assortment of cells^55–57^, we then analyzed whether the spheroids consist of different cell types. Therefore, sections of JB4 embedded spheroids were stained with hematoxylin and eosin (H&E) to provide an overview of the spheroid structure and morphology as well as to identify the possible existence of cell types other than adipocytes (Fig. 5a). Strikingly, small, dense cells could be detected, indicating that spheroids were not only composed of differentiated adipocytes, but may contain other cell types, such as immune cells. In particular, macrophages play an important role in the regulation of adipose tissue metabolism in lean and obese organisms^36,58^. While culturing the SVF in as a 2D monolayer, it was observed that SVF macrophages could only be maintained upon addition of M-CSF to cell culture medium, as shown by F4/80 staining (Fig. 5b). Interestingly, in differentiated 3D spheroids, resident SVF macrophages could be retained without M-CSF supplementation in the culture medium (Fig. 5c). By immunofluorescence microscopy, F4/80-positive cells could clearly be identified within spheroids. To corroborate this finding, the cell composition of the spheroids was also analyzed by flow cytometry. Approximately 20–30% of all spheroid cells were CD45+ immune cells (Fig. 5d, Supplementary Fig. S2). Of the immune cell population, 60–70% were CD11b+F4/80+ macrophages. Thus, growth of spheroids in a 3D environment enables the maintenance of SVF-derived macrophages without the addition of any external macrophage growth factors. In addition, we could show that undifferentiated spheroids produced high amounts of M-CSF (Fig. 5f), which may favor the maintenance of macrophages in the spheroid. However, fully mature spheroids, did not produce M-CSF, which might be in line with the observation that the macrophage number does not increase during spheroid differentiation. It was also evident that 30–40% of CD45+ immune cells were negative for the myeloid cell marker CD11b (Fig. 5d). Recently, mast cells were identified in human SVF-derived spheroids^59^. In line, approximately 10% of FcεR1+ckit+cells, presumably mast cells, were observed also in our spheroids (Fig. 5d, right panel). In addition, single cell suspensions from spheroids were analysed for the presence of CD45− CD31+ endothelial cells. However, no endothelial cells could be detected in the spheroids (Supplementary Fig. S2). To clarify whether macrophages were present in spheroids prior, during and after adipocyte differentiation, flow cytometry analysis was performed throughout the differentiation process (Fig. 5e). Undifferentiated spheroids which had been seeded into ULA plates for 24 h were found to already contain a population of CD11b+F4/80+ macrophages. Interestingly, the amount of CD45+ immune cells increased during spheroid differentiation from approximately 14% in undifferentiated cells to approximately 25–30% upon differentiation. In contrast, the proportion of macrophages within the CD45+ immune cell population as well as the proportion of mast cells remained constant during the differentiation process, indicating that populations of other cells types expand in spheroids during maturation (Fig. 5e).

### LPS- and IL-4-induced changes in the spheroid lipidome

**Figure 6:**
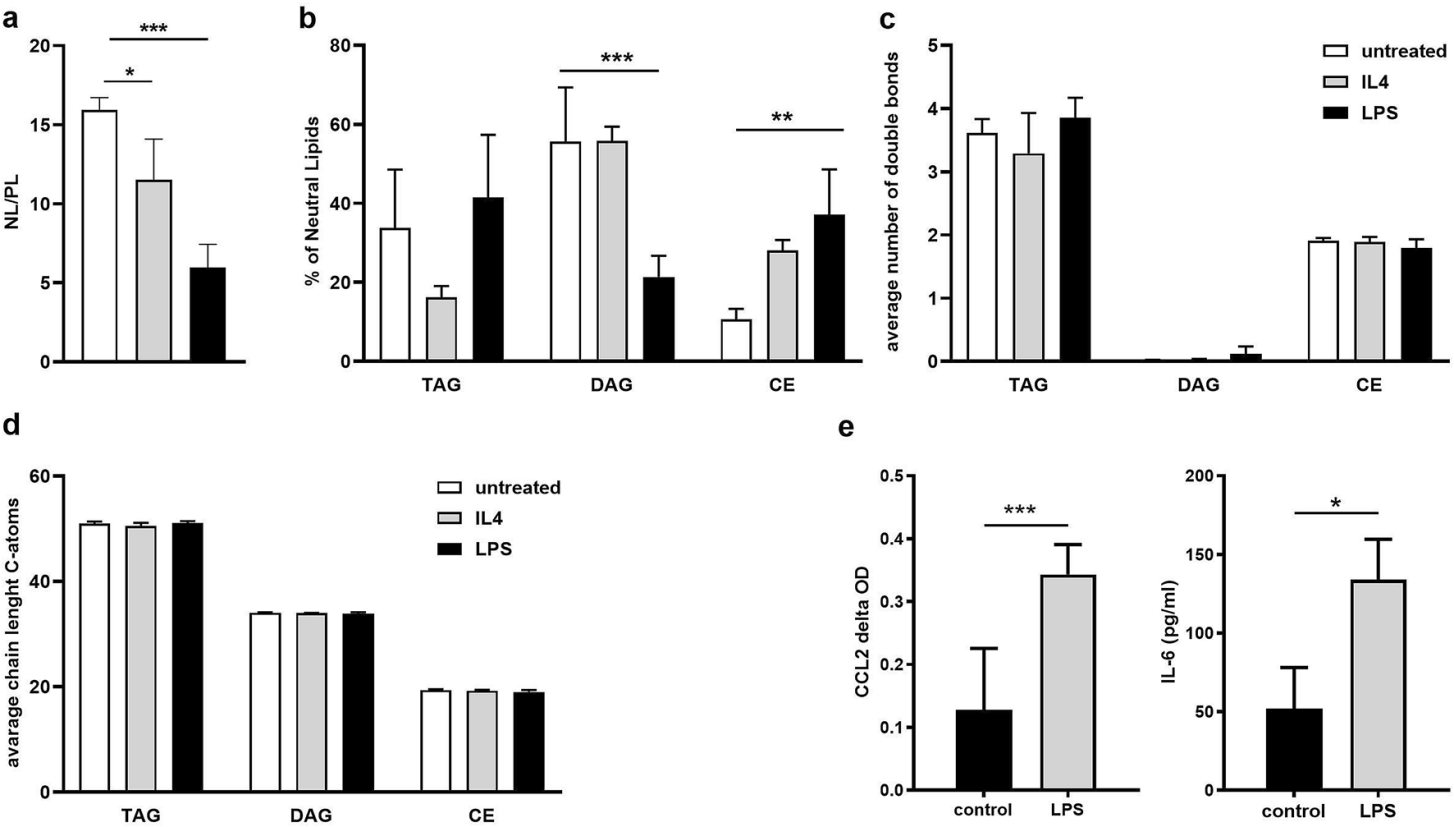
LPS- and IL-4-treatment induced changes in the organoid lipidome. SVF were differentiated, cultivated with Insulin for 14 more days before treated with IL-4 or LPS for 1 h. Total lipids were extracted and analysed by MS. (**a**) Ratio of neutral lipids to polar lipids. (**b**) composition of neutral lipids. (**c**) Average number of double bonds. (**d**) Average chain lengths of neutral lipids (n = 3 individual experiments in triplicates). (**e**) Induction of pro-inflammatory CCL2 and IL-6 levels after LPS stimulation (n = 3 individual experiments (biological replicates) each performed in technical triplicates). Data are presented as mean ± SD. Significance was determined using two way ANOVA with Dunette correction for multiple comparisons. *p < 0.05, **p < 0.01 and ***p < 0.001.

Obesity is characterized by a chronic low grade inflammation which is driven by a reciprocal interaction between adipose tissue macrophages and adipocytes^36^. Therefore, we aimed to study the interaction between inflammatory signaling and lipid metabolism. For this, we chose to compare pro- and anti-inflammatory environments by treating spheroids either with LPS, to create a pro-inflammatory environment, or IL-4, which acts to induce anti-inflammation^60,61^ but also interferes with lipid metabolism^62^. Spheroids were treated from day 9 of differentiation for 14 days with insulin. Thereafter, spheroids were stimulated for 1 h with LPS or IL-4. Lipids were isolated and analyzed by MS to monitor lipidomic changes. For treatments with either immune regulator, the ratio of NL-to-PL was reduced compared to the untreated control (Fig. 6a) with a stronger response to LPS than to IL-4 treatment. The frequencies of NL species differed substantially after treatment with IL-4 or LPS (Fig. 6b). After IL-4 treatment, TAG frequencies decreased, while CE frequencies increased. DAG levels remained unchanged. In LPS-treated spheroids, TAG fractions remained unaltered, however, a decrease in DAG and increase CE fractions were visible (Fig. 6b). Despite this observation, no changes could be observed in the average carbon chain length and in the amount of double bonds in NL after either IL-4 or LPS treatment (Fig. 6c,d). A more detailed analysis of TAG, DAG and CE species is also depicted as heat maps (Supplementary Fig. S3). To further confirm that LPS treatment was able to induce an inflammatory state in spheroids, the secretion of pro-inflammatory cytokines was assessed. LPS stimulation increased both the expression of the pro-inflammatory cytokine IL-6 as well as the obesity-induced chemokine, C–C Motif Chemokine Ligand 2 (CCL2) (Fig. 6e). Thus, adipose tissue spheroids can be used to monitor metabolic alterations induced by pro- or anti-inflammatory signals by MS-based lipidomics.

## Discussion

Here we developed a SVF-based 3D adipose tissue organoid model composed of different cell types, including mature adipocytes, macrophages and mast cells. Using this model, we could demonstrate that lipidomic changes during differentiation or upon treatment with dietary or inflammatory factors can be monitored by MS-based lipidomics.

We and others used SVF cells to generate scaffold-free 3D spheroids^12,13,16^. Using the scaffold free approach, spheroids consisting of mature adipocytes as well as other cells types can be generated. Besides mature adipocytes, spheroids were confirmed to also contain immune cell populations. A recent paper identified a mast cell population that ‘fell out’ and grew underneath SVF-derived spheroids seeded in ULA plates^59^. Here, we also observed that mast cells were retained within spheroids. Macrophages comprise the most abundant immune cell type in adipose tissue and are necessary for adipocyte differentiation and tissue maintenance^36^. In addition, they are major players in the development of obesity^63–65^. While F4/80+ macrophages in 2D SVF monolayers were only detectable after the addition of M-CSF, macrophages in 3D spheroid cultures were found to be integrated within spheroids without the addition of growth factors. The maintenance of macrophages within the spheroids is likely explained by the expression of M-CSF by undifferentiated adipocytes, which is not just a macrophage colony stimulating factor but also plays a role in adipose tissue growth^66^. Although we were able to identify macrophages and mast cells as part of the spheroid immune cell population, we were not able to detect endothelial cells. This may be due to a lack of endothelial growth factors, such as VEGF, in cell culture medium, which are required to support the maintenance and proliferation of endothelial cells. In a recent study, Muller et al. also showed that culturing human SVF cells in the standard adipocyte differentiation cocktail led to the loss of endothelial cells in spheroids. However, by culturing the SVF in an endothelial growth medium containing various growth factors followed by embedding spheroids in matrigel supplemented with BMP7 and fatty acids, they were able to maintain endothelial cells and vascular networks while inducing adipocyte differentiation. Therefore, variations of the standard differentiation cocktail as well as culture conditions may favor the growth of different cell populations of the SVF in a 3D environment. We were able to classify the spheroids generated by the methods used within this study as organoids^13^. Not only were cells able to self-organize into a tissue containing several different cell types, spheroids were also demonstrated to execute specialized functions which resemble the in vivo organ. In this case, storage of fat and the induction of cytokine expression following treatment with inflammatory stimuli were used as an example. The opportunity to maintain resident adipose tissue macrophage and mast cell populations within organoid cultures offers the ability to better understand the role of the immune system in adipose tissue biology. Since inflammation is mainly responsible for obesity-associated complications such as diabetes as well as obesity-associated cancer development^5,6^, this model proves to be a useful tool to study various adipose tissue related diseases.

The main function of adipose tissue is the storage of energy in the form of neutral lipids packaged in lipid droplets. Induction of adipocyte differentiation led to spheroid enlargement, presumably due to the formation of large unilocular lipid droplets, as previously reported^12^. The ability of spheroid adipocytes to acquire monolocular rather than multilocular lipid droplets is also reflected by the differences in lipid composition between organoids and monolayer cultures. By applying mass spectrometry-based lipidomics to our 3D model, this allows for the investigation of metabolic changes in in vitro cultured adipose tissue. One advantage of using this system is the possibility to work in a 96 well format in which little cellular material is required. This enables the investigation of multiple different conditions in a high-throughput manner and opens the doors for large scale therapeutic studies. The lipidomic profiles of 2D and 3D cultures compared to in vivo adipose tissue revealed that the spheroid lipidome is more comparable to in vivo adipose tissue compared to 2D monolayers, thus reflecting the physiological relevance of the 3D system. Additionally, we observed changes in the lipidome during the adipocyte differentiation process. We performed a detailed analysis of the composition of total and specific neutral lipid species during differentiation. Most prominently, we observed an increase in the ratio of neutral lipids which resulted in a decrease in phospholipids during adipocyte development. This was reflected by the synthesis and expansion of unilocular lipid droplets in 3D cultures. How such alterations in the ratio of membrane lipid species could potentially alter cellular function remains a major biological question^67^. Lipidomic changes during the adipocyte differentiation process were also observed by Miehle et al., who followed lipidomic alterations during adipogenic differentiation of the SGBS cell line. They observed a more substantial increase in the NL fraction in 2D cell cultures compared to our primary SVF-based system, however these differences could be caused by the use of different differentiation protocols and the use of human cell lines versus primary murine cells in our system^68^. We then evaluated changes in the lipidome of the organoids by treating them with fatty acids or insulin. Fatty acid feeding of organoids induced changes in the ratios of different TAG species. Therefore, we could demonstrate that our organoid model is a useful tool in tracking lipidomic changes in response to dietary interventions. In adipose tissue, insulin induces adipocyte hypertrophy through the induction of lipogenesis and inhibition of lipolysis^69^. Here, increases in total neutral lipid content and more subtle changes in individual neutral lipid species were observed in 3D organoids after insulin treatment, indicating that 3D models are more appropriate to investigate the consequences of insulin-induced signaling. Keeping into consideration Huynh et al. have previously shown that fatty acid treatment can lead to macrophage lipidomic changes, thus, lipidomic changes in the organoid do not only reflect changes in the adipocyte lipidome, but also reflect lipidomic changes of all cell populations found within the organoids. Therefore, organoid lipidomic changes more closely reflect changes in adipose tissue in response to stimuli compared to 2D cultured monolayers^70^. It would be interesting in the future to subject spheroids to conditions which resemble obese adipose tissue to be used for diabetes or obesity studies, such as by long term insulin treatment or high glucose and fatty acid levels. These organoids can then be analyzed for their metabolic change or changes in gene expression of metabolic and inflammatory target genes^71^.

Furthermore, we investigated spheroid lipid metabolism under immunomodulatory conditions. Insulin resistance is one of the primary causes of type 2 diabetes. IL-4 is known to promote insulin sensitivity^62^, induce lipolysis^72^ and regulate lipid metabolism. Similar to in vivo, IL-4 led to a decrease in spheroid neutral lipid content^62^. Type 2 diabetes and metabolic syndrome are considered as inflammatory chronic diseases. Both adipocytes and adipose tissue immune cells can react to inflammatory stimuli^58^. Dermal adipocytes can also respond to bacterial infections through the production of antimicrobial peptides, indicating that adipose tissue is important to regulate host defense and inflammation^73^. Stimulation of spheroids with LPS induced pro-inflammatory cytokine and chemokine secretion, including IL-6 and CCL2. Pro-inflammatory cytokines are key contributors to lipid spillover observed in obesity and other metabolic disorders by switching on lipolysis^74–76^. In accordance, LPS treatment of spheroids also induced a reduction in the levels of neutral lipids, fitting the concept that a pro-inflammatory microenvironment stimulates adipocyte lipolysis^77–79^. When challenged with pro-inflammatory stimuli, a cytokine response was evoked in organoids that was shown to impact lipid metabolism. Therefore, we propose this model is suitable for immune-metabolic research.

In conclusion, we have successfully grown an adipose tissue organoid model which contains mast cells and macrophages without the need for addition of external growth factors and has been proven to be suitable for metabolic studies. This model will be helpful to investigate challenging questions regarding development and function of adipose tissue as well as for the investigation of pathological conditions such as obesity or diabetes.

## Materials and methods

### Isolation and culture of adipose stromal vascular fraction

Healthy, lean, 7–9 weeks old male C57BL/6 NCrl mice with a weight of approximately 24–29 g were killed by cervical dislocation to excise visceral fat. The isolated adipose tissue was washed with phosphate-buffered saline (PBS) containing 5 μg/mL amphotericin B, minced thoroughly and digested in PBS containing 0.4 U/mL Liberase (Roche) and 5 μg/mL amphotericin (Life technologies). The digested tissue was filtered through a 100 μm cell strainer, diluted 1:2 with growth medium [Dulbecco’s Modified Eagle’s medium low glucose (DMEM) supplemented with GlutaMAX (Life Technologies), 20% Fetal Calf Serum (FCS), 1% penicillin/streptomycin (P/S) and 5 μg/mL amphotericin B] and centrifuged. The remaining pellet was resuspended in growth medium [Dulbecco’s Modified Eagle’s medium (DMEM) supplemented with GlutaMAX (Life Technologies), 20% FCS, 1% P/S] and seeded into cell culture dishes. Medium was changed the next day to DMEM supplemented with GlutaMAX containing 20% FCS without the addition of P/S or amphotericin B. Cells were incubated at 37 °C and 5% CO_2_ until 80—100% confluency. 50,000 cells per well in 100 µL were seeded in Nunclon Sphera 96-well ULA U-bottom plates (Thermo Fischer). All mouse experiments were performed according to German and Institutional guidelines for animal well fare and experimentation and were approved by the local authorities of North Rhine-Westphalia (Germany).

### Adipose differentiation of spheroids

48 h after seeding SVF in ULA plates, spheroids were formed and medium was changed to induction medium (DMEM with GlutaMAX, 20% FCS, 5 μg/mL insulin, 0.5 mM 3-isobutyl-1-methylxanthin (IBMX), 1 μM dexamethasone and 4 μg/mL rosiglitazone. 48 h later, induction medium was replaced by differentiation medium (DMEM with GlutaMAX, 20% FCS, 5 μg/mL insulin) and replaced by fresh differentiation medium at day 6. At day 8 spheroids were transferred in growth medium (DMEM with GlutaMAX, no insulin). At day 9 they were considered as fully differentiated and were cultured further, with medium changes every 3 days. For fatty acid feeding, they were treated from day 9 on every 48 h with 0.5 mM OA or 0.5 mM SA or left in growth medium without additives for 14 additional days and lipid metabolism was examined by MS thereafter. For inflammation experiments, spheroids were treated from day 9 of differentiation for 14 days with insulin. After that, they were stimulated for 1 h with LPS (100 ng/mL) or IL-4 (20 ng/mL), lipids were isolated and analyzed by MS.

### Calculation of spheroid volume

Assessment of relative increase in spheroid volume during adipogenic differentiation was enabled by the application of microscopy in combination with a high-throughput CellProfiler pipeline. Spheroids plated in a 96-well ultra-low attachment U-bottom plate were microscoped using a Zeiss Axio Observer Z1 with 20 × LD Plan-Neofluar. Four brightfield channel picture tiles were taken per central region of each well and stitched together with 10% overlap and pixels binned 2 × 2 resulting in increased brightness and reduced data size. Using the Zeiss Zen software’s SplitScenes function, image stacks were separated and then further analyzed with the CellProfiler Analyst software developed by Whitehead and Broad Institute starting by inversion of the brightfield channel by the ImageMath package^45^. Afterwards, spheroids were identified using the IdentifyPrimaryObjects package. An adaptive Otsu algorithm-based two-class threshold was applied with a correction factor of 1 and an adaptive window of 200 pixels. Smoothing and declumping were not performed, but cavities inside the defined object were replenished. Images without detectable objects were again assessed using smoothing equivalent to one sigma. The size parameters of the defined objects were assessed by using the MeasureObjectSizeShape package. Therefore, an ellipse with the same second moments as the object was created to allow for extraction of the minor axis length as well as the area of the whole object into a spreadsheet. Assuming the form of a prolated spheroid for the spheroids, volumes were calculated with the formula *V* = 4/3*A*a, whereas the halved minimal diameter of the fitted ellipse is used as *a* and the complete threshold defined area as *A*. Due to contraction of the spheroids upon plating and different plated cell counts, calculated spheroid volumes were normalized against the volume determined 2 days after plating to assess reliable relative changes in spheroid volume.

### Immunohistochemistry

Spheroids were washed once with PBS, stained with 1 μg/mL of DAPI and 0.5 µg/mL LD540 in PBS^80^ or actin green according to manufacturer’s instructions (Molecular probes) for 15 min. Spheroids were washed twice with PBS. Spheroids were embedded in PBS or Fluoromount G embedding medium, with cover slips as spacers to avoid crushing of the specimen. LSM image acquisition was done with an inverse Zeiss LSM710 with standard equipment. Image processing was done with Fiji/Image J and Adobe Photoshop software.

### JB4 embedding and H&E staining

For embedding the JB4 Embedding Kit of Sigma-Aldrich was used by following the instructions. H&E staining was performed on 5 µm thick microtome sections.

### Lipidomics

Lipidomic analysis was done as described^81^. Briefly, 500 μL of extraction mix [CHCl_3_/MeOH 1/5 containing internal standards: 210 pmol PE (31:1), 396 pmol PC (31:1), 98 pmol PS (31:1), 84 pmol PI (34:0), 56 pmol PA (31:1), 51 pmol PG (28:0), 28 pmol CL (56:0), 39 pmol LPA (17:0), 35 pmol LPC (17:1), 38 pmol LPE (17:0), 32 pmol Cer (17:0), 99 pmol SM (17:0), 55 pmol GlcCer (12:0), 14 pmol GM3 (18:0-D3), 359 pmol TG (47:1), 111 pmol CE (17:1), 64 pmol DG (31:1), 103 pmol MG (17:1), 724 pmol Chol (d6), and 45 pmol Acyl-Car (15:0)] were added, and sonicated for 2 min. The material was collected into a tube followed by centrifugation at 20,000*g* for 2 min. The supernatant was transferred into a fresh tube and 200 μL chloroform and 800 μL 1% AcOH in water were added, and the sample was briefly shaken and spun for 2 min at 20,000*g*. The upper aqueous phase was removed and the entire lower phase transferred into a new tube and evaporated in the speed vac (45 °C, 10 min). Spray buffer (500 μL of 8/5/1 2-propanol/MeOH/water and 10 mM ammonium acetate) was added, the sample sonicated for 5 min, and infused at 10 μL/min into a Thermo Q Exactive Plus spectrometer equipped with the HESI II ion source for shotgun lipidomics. MS1 spectra (resolution 280,000) were recorded in 100 m/z windows from 250 to 1200 m/z (pos.) and 200 to 1700 m/z (neg.) followed by recording MS/MS spectra (res. 70,000) by data-independent acquisition in 1 m/z windows from 200 to 1200 (pos.) and 200 to 1700 (neg.) m/z. Raw files were converted to .mzml files and imported into and analyzed by LipidXplorer software using custom mfql files to identify sample lipids and internal standards. For further data processing, absolute amounts were calculated using the internal standard intensities followed by calculation of mol% of the identified lipids^81^.

### Flow cytometry

Three spheroids were centrifuged for 3 min at 500 g and washed once with PBS, collected and digested in 4U/mL Liberase (Roche) for 20 min. Cell suspensions were stained with antibodies against CD45 (30-F11, Biolegend), F4/80 (BM8, Biolegend), CD11b (M1/70, eBioscience), CD31 (Mec 13.3, Biolegend), CD117/c-kit (2B8, eBioscience), FcεR1 (MAR-1, eBiocsience). Cell populations were recorded with a FACSCanto II flow cytometer (BD Biosciences); data were analyzed with FlowJo software (Tree star, Ashland, USA).

### ELISA

Three spheroids per well were pooled in a 96-well ULA plate and stimulated with 100 ng/mL LPS in 200 µL complete low glucose DMEM for 16 h. Cytokine concentrations in cell culture supernatants were measured by ELISA specific for IL-6 (Bio-Techne, Minneapolis, USA) and CCL2 (Peprotech, Hamburg, Germany). M-CSF was measured in the supernatants of single spheroids by ELISA (Peprotech, Hamburg, Germany).

### Data analysis

Data are presented as mean ± SD. Data were analyzed with Graphpad Prism software using unpaired Student's two-tailed t test or Two Way ANOVA with Dunette correction for multiple comparisons. Significance levels were denoted in accordance to their p value as *for < 0.05, **for < 0.01 and ***for < 0.001.

## Supplementary information

Supplementary Information.
