# Supplementary material for: Generation of immune cell containing adipose organoids for in vitro analysis of immune metabolism
Source: Sci Rep. 2020 Dec 3;10:21104. doi: 10.1038/s41598-020-78015-9 (PMC7713299; doi:10.1038/s41598-020-78015-9)
Supplement: Supplementary file 1 — Supplementary Information. [file 41598_2020_78015_MOESM1_ESM.pdf]

Generation of immune cell containing adipose organoids for in vitro analysis of immune metabolism

Jacqueline Taylor[1, 2, 3], Julia Sellin[4], Lars Kuerschner[1], Lennart Krähl[5], Yasmin Majlesain[5], Irmgard Förster[5], Christoph Thiele[1], Heike Weighardt[*, 5] and Elvira Weber[**, 1]

[1] Biochemistry & Cell Biology of Lipids, Life and Medical Sciences Institute (LIMES), University of Bonn, Carl-Troll-Straße 31, D-53115, Bonn, Germany.

[2] present address: Division Vascular Signaling and Cancer (A270), German Cancer Research Center (DKFZ), 69120 Heidelberg, Germany.

[3] present address: Faculty of Biosciences, University of Heidelberg, 69120 Heidelberg, Germany

[4] ZSEB, Center for Rare Diseases, University Hospital Bonn, 53127 Bonn, Germany

[5] Immunology & Environment, Life and Medical Sciences (LIMES) Institute, University of Bonn, Carl-Troll-Straße 31, D-53115, Bonn, Germany.

Running title: 3D organoids for lipidomic analyses

*corresponding author: 0049 228 73 62706, email: heike.weighardt@uni-bonn.de
**corresponding author: 0049 178 8952025, email: eweber1@uni-bonn.de

Supplementary Figure 1

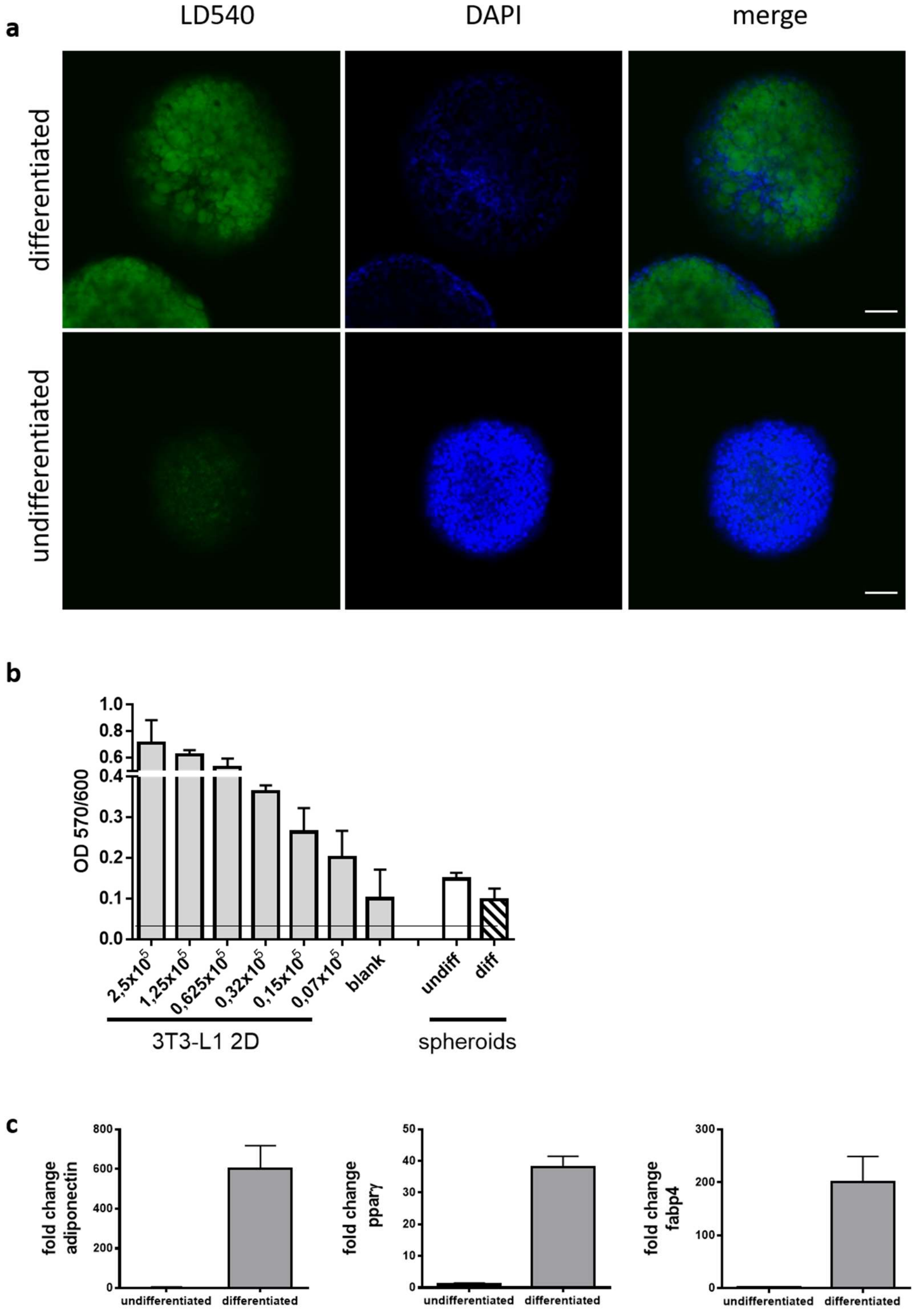

Figure S1: Lipid droplet accumulation and enhanced expression of adipose marker genes in differentiated spheroids

a) Spheroids were washed with PBS and fixed in 1% PFA in PBS for 30 min. After washing with PBS 1% Triton, spheroids were stained with 0.2 µg/mL DAPI and 0.125 µg/ml LD540 in PBS 1% Triton for 4 h. After washing in PBS, spheroids were mounted in Aqua-Poly/Mount (Polysciences). Images were acquired using a ZEISS LSM 880 Laser scanning microscope equipped with an objective LCI Plan Neofluar 10x/0.3 M27 and ZEN software. Image processing was done with Fiji/Image J. Representative images are shown. b) AlamarBlue assay for proliferation analysis. 4 organoids were pooled in 200 µl culture medium, supplemented with 20 µl AlamarBlue solution (Biorad) and incubated for 6 h at 37 °C, 5 %CO_2. Absorbance was measured at 570 nm and 600 nm. As a control different amounts of 3T3-L1 cells were seeded in parallel to the spheroids and were included in the assay (n=2 experiments with 4 biological replicates of organoids). c) 5-7 spheroids were collected per sample and digested in 0.4U/ml Liberase (Roche) for 15min. After vortexing, cells were immediately mixed with Trizol. RNA was prepared using the Direct-zol RNA Microprep Kit (Zymo research, Freiburg, Germany) according to the manufacturer's instruction. cDNA was synthesized using Revert Aid reverse transcriptase (Thermo Fisher Scientific, Bonn, Germany). Real-time PCR was performed on a BioRad CFX96 Real-Time PCR Detection System using absolute SYBR-green ROX master mix (Thermo Fisher Scientific). Primers were designed using the Universal Probe Library (Roche Applied Science, Mannheim, Germany): pparγ fwd: 5′-ggaagaccactcgcattcctt-3′, pparγ rev: 5′-gtaatcagcaaccattgggtca-3′, adiponectin fwd: 5′-ggagagaaaggagatgcaggt-3 ′, adiponectin rev: 5′-ctttcctgccaggggttc-3′, fabp4 fwd: 5′-ggatggaaagtcgaccacaa-3 ′, fabp4 rev: 5′-tggaagtcacgcctttcata -3′, β-actin fwd: 5′-ctaaggccaaccgtgaaaag-3 ′, β-actin rev: 5′-accagaggcatacagggaca-3′.(n= 6-7 biological replicates of organoids).

Supplementary Figure 2

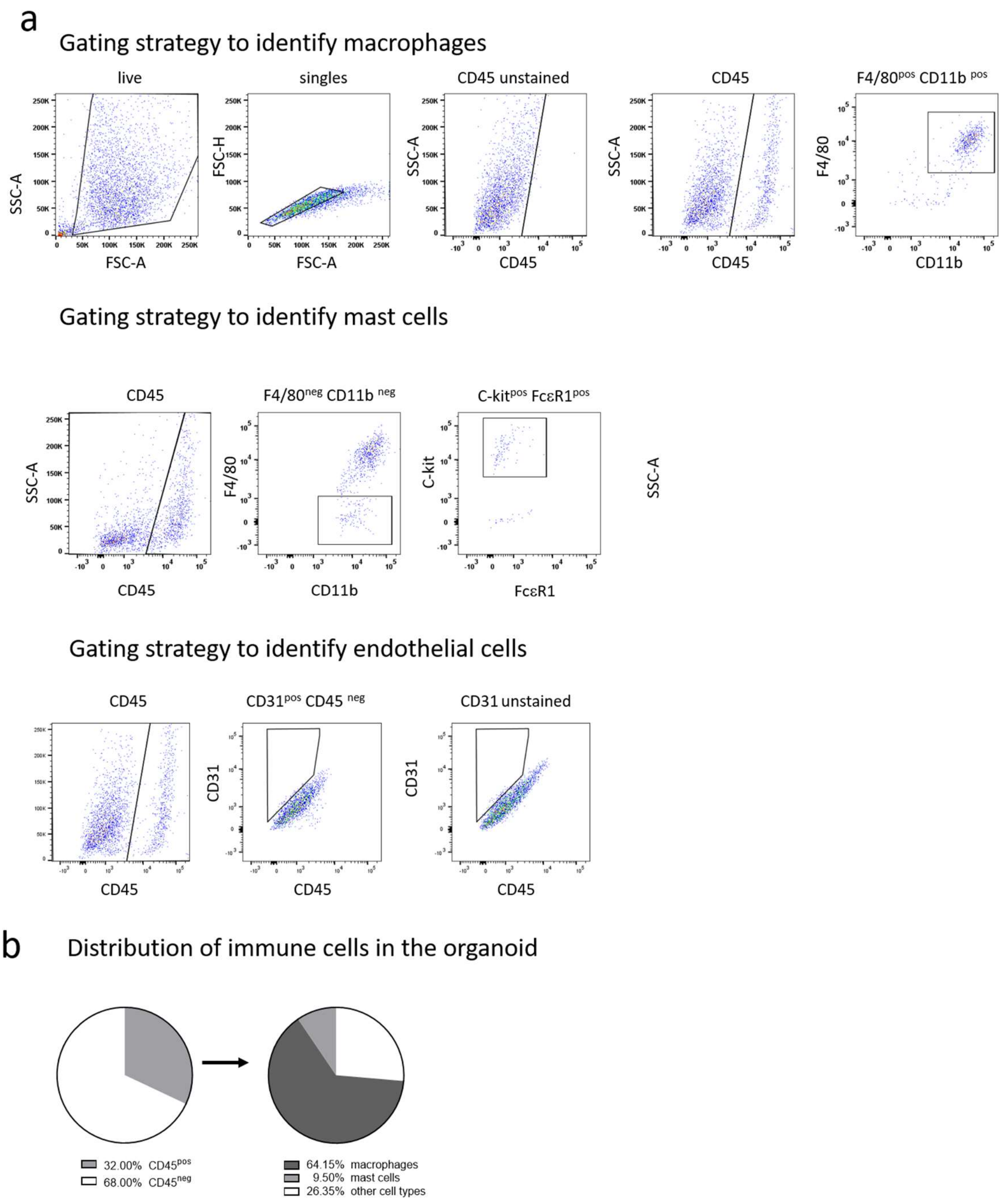

Figure S2: FACS analysis of spheroids

(a) Gating strategies to identify macrophages, mast cells and endothelial cells. (b) Mean distribution of CD45 positive and CD45 negative cells in single cell suspensions of the spheroid. Mean frequencies of macrophages and mast cells within the CD45 positive fraction are shown (n=3-6 independent experiments in triplicates).

Supplementary Figure 3

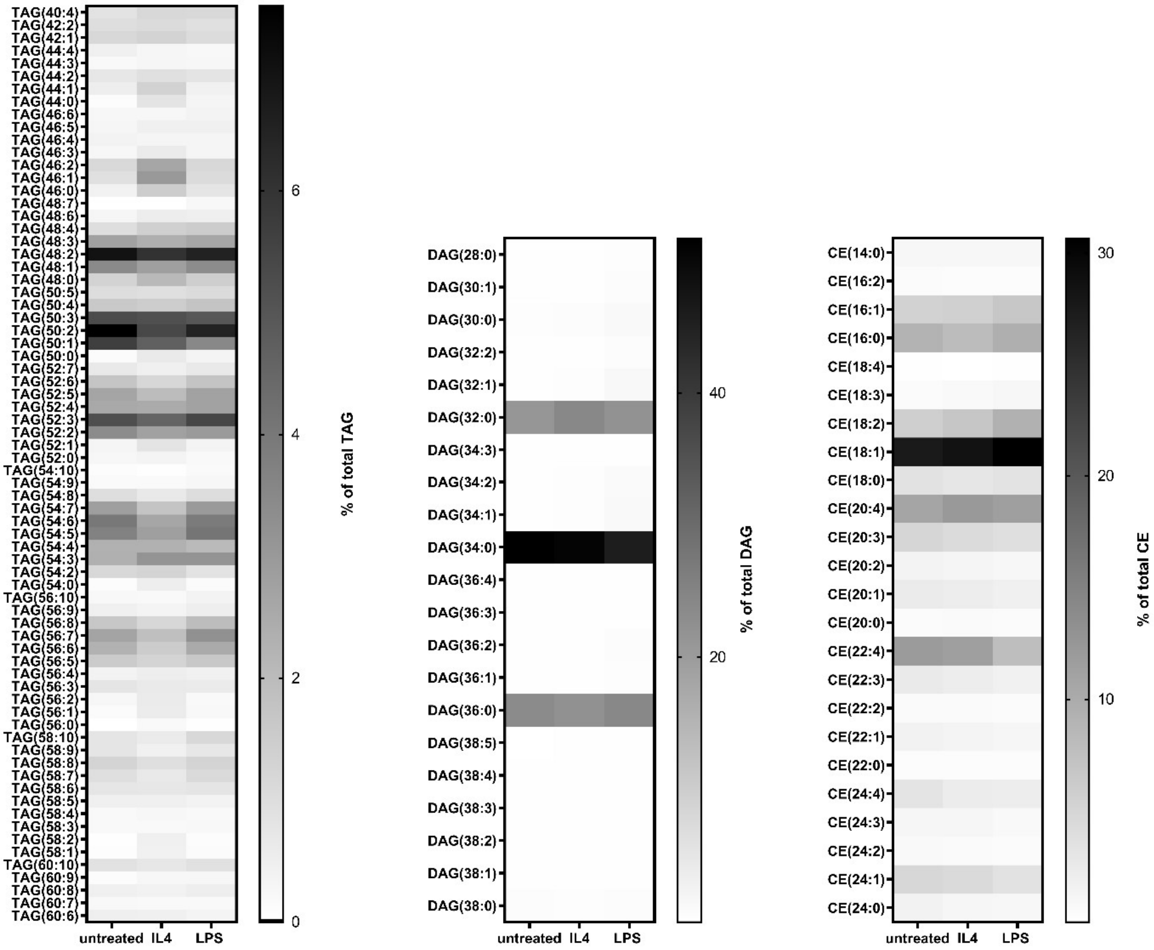

Figure S3: LPS- and IL-4-treatment induced changes in the organoid lipidome.

SVF were differentiated, cultivated with insulin for 14 more days before treated with IL-4 or LPS for 1 hour. Total lipids were extracted and analyzed by MS. TAG, DAG and CE species were presented as a heat map. (n=3 independent experiments in triplicates).
